# Enhanced l-ornithine production from glucose and sucrose via manipulation of the fructose metabolic pathway in *Corynebacterium glutamicum*

**DOI:** 10.1186/s40643-022-00503-9

**Published:** 2022-02-08

**Authors:** Libin Nie, Kexin Xu, Bin Zhong, Xiaoyu Wu, Zhongtao Ding, Xuelan Chen, Bin Zhang

**Affiliations:** 1 College of Bioscience and Bioengineering, Jiangxi Engineering Laboratory for the Development and Utilization of Agricultural Microbial Resources, Jiangxi Agricultural University, Nanchang, 330045 China; 2 College of Life Science, Jiangxi Normal University, Nanchang, 330022 China

**Keywords:** Green biomanufacturing, *Corynebacterium glutamicum*, l-Ornithine, Sucrose

## Abstract

l-Ornithine, an important non-essential amino acid, has considerable medicinal value in the treatment of complex liver diseases. Microbial fermentation strategies using robust engineered strains have remarkable potential for producing l-ornithine. We showed that glucose and sucrose co-utilization accumulate more l-ornithine in *Corynebacterium glutamicum* than glucose alone. Further manipulating the expression of intracellular fructose-1-phosphate kinase through the deletion of *pfkB1*resulted in the engineered strain *C. glutamicum* SO30 that produced 47.6 g/L of l-ornithine, which represents a 32.8% increase than the original strain *C. glutamicum* SO26 using glucose as substrate (35.88 g/L). Moreover, fed-batch cultivation of *C. glutamicum* SO30 in 5-L fermenters produced 78.0 g/L of l-ornithine, which was a 78.9% increase in yield compared with that produced by *C. glutamicum* SO26. These results showed that manipulating the fructose metabolic pathway increases l-ornithine accumulation and provides a reference for developing *C. glutamicum* to produce valuable metabolites.

**Graphical Abstract:**

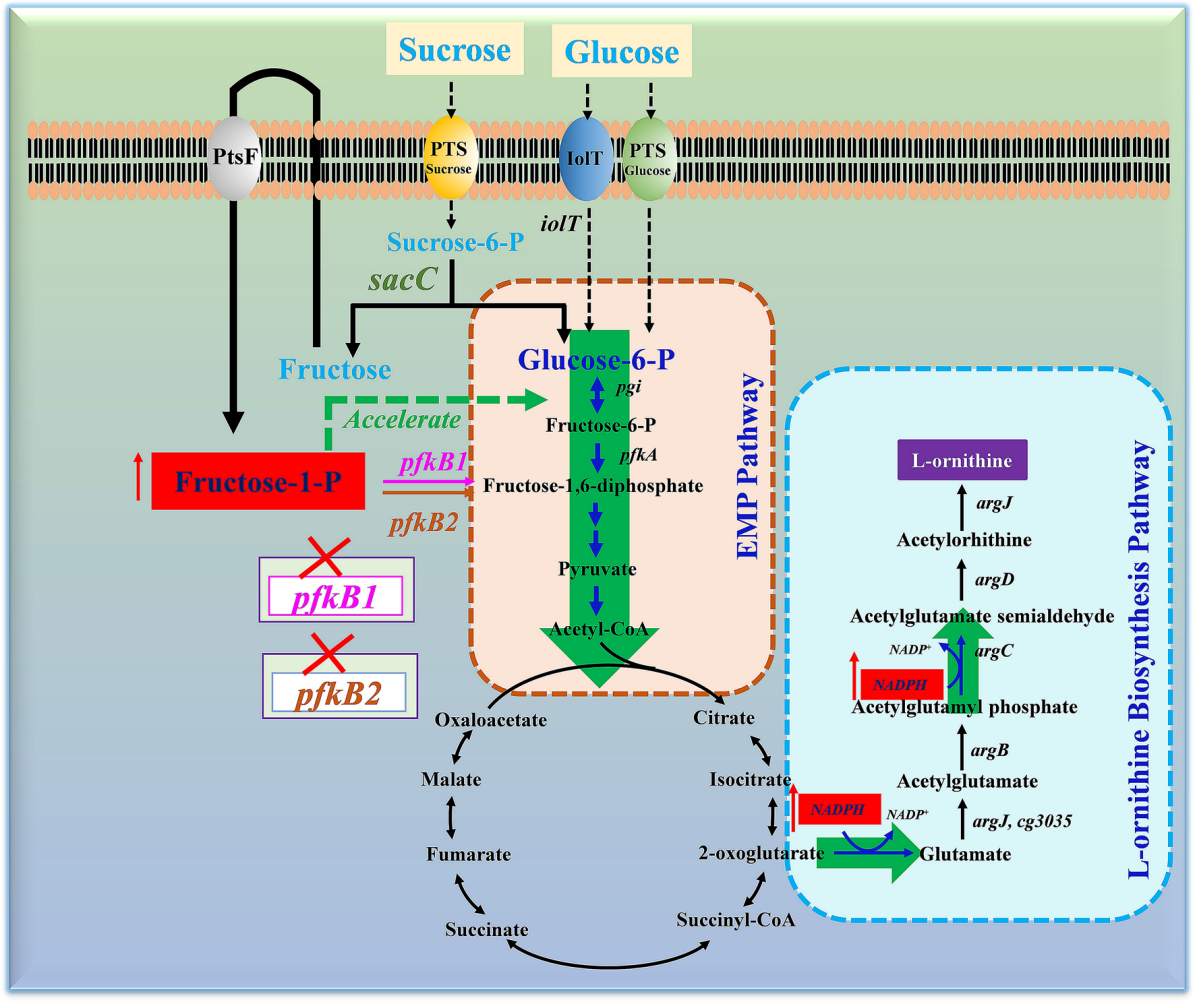

## Introduction

l-Ornithine, an important non-essential amino acid of significant medicinal and economic value in the treatment of complex liver diseases, is widely applied as a food additive and chemical pharmaceutical intermediate (Jover-Cobos et al. 2014). Enormous demands for these have led to imminent requirements for increased production capacity. The microbial fermentation approach has considerable potential for l-ornithine production, which requires robust engineered strains to reduce costs. Therefore, effective strategies are needed to construct such strains that can produce abundant l-ornithine.

*Escherichia coli* (Lee and Cho 2006), *Saccharomyces cerevisiae* (Qin et al. 2015), and *Corynebacterium glutamicum* (Wu et al. 2020) have been engineered to produce l-ornithine using metabolic engineering strategies. Among these, *C. glutamicum* is an established industrial workhorse and the principal microbial cell factory for generating strains that can produce l-ornithine because it does not produce endotoxin, grows rapidly, and gene manipulation is simple (Becker and Wittmann 2015; Mitsuhashi 2014). Thus, increasing l-ornithine production titers has been extensively studied in *C. glutamicum*. This has resulted in several engineered *C. glutamicum* strains being created by knocking out genes in competing pathways, improving precursor availability, deregulating feedback loops, increasing intracellular cofactor availability, developing high-affinity transport systems, and dredging carbon transportation and metabolic pathways (Wu et al. 2020). Notably, *C. glutamicum* KBJ11 with optimal l-ornithine production performance was engineered via the CRISPR-Cpf1-based inactivation of *argF*, *argR*, and *ncgl2228*. Overexpressed *CsgapC* and *BsrocG*, which participate in l-ornithine production, led to the generation of 88.26 g/L and a yield of 0.414 g/g glucose during 72 h of fed-batch cultivation (Dong et al. 2020). Although this yield of l-ornithine has potential industrial value, a large gap remains between the production titer and the theoretical maximum yield. Genetic modification strategies primarily focus on obvious direct modification targets. However, indirect, and hidden targets, which have not yet been investigated, also play important roles in l-ornithine biosynthesis. For instance, the positive synergistic effect of sucrose and glucose on improving l-arginine production is not mentioned in breeding l-ornithine-producing strains (Park et al. 2014).

Sucrose extracted from sugarcane is second to glucose as the most abundant carbon source, and it is intensively applied in industrial fermentation (Georgi et al. 2005; Blombach and Seibold 2010; Zhang et al. 2020a). Numerous microorganisms can utilize sucrose as a carbon source to synthesize valuable metabolites (Zhang et al. 2018c, 2018e; Zhao et al. 2020). After intracellular transport and subsequent phosphorylation, sucrose can be converted to glucose phosphate and fructose by sucrose-6-phosphate hydrolase in *C. glutamicum* (Zhang et al. 2015b). Sucrose uptake is processed through a type II phosphotransferase system coupled with sugar phosphorylation (Martins et al. 2019). Sugar phosphates are then further metabolized in the glycolysis pathway through the fructose-1,6-diphosphate metabolic node. Using this metabolic pathway, sucrose has been utilized as an attractive substrate for *C. glutamicum* to produce l-serine (Zhang et al. 2020b, 2018d), l-lysine (Sgobba et al. 2018; Xu et al. 2020), l-pipecolic acid (Pérez-García et al. 2017), l-arginine (Park et al. 2014), scyllo-inositol (Ramp et al. 2021), and shikimic acid (Zhang et al. 2015a). Sucrose as a carbon source can be superior to glucose in terms of accumulating chemicals. For instance, the highest l-serine titer produced from sucrose by *C. glutamicum* SSAAI-serE was 43.9 g/L, which is significantly higher than that obtained with glucose as the substrate (Zhang et al. 2020b). In addition, the intracellular concentrations of the cofactor nicotinamide adenine dinucleotide phosphate (NADPH), fructose phosphate, and glucose phosphate are remarkably improved when *C. glutamicum* was cultivated with sucrose as the substrate (Wang et al. 2016). Increased supplementation with intracellular NADPH promotes l-lysine and l-arginine accumulation (Wang et al. 2016). The simultaneous biosynthesis of fructose phosphate and glucose phosphate remarkably stimulates the central metabolic pathway, improving the yield of valuable metabolites (Hasegawa et al. 2017). Therefore, mixtures of glucose and sucrose have been used to improve the production titers of l-arginine and l-pipecolic acid (Park et al. 2014; Pérez-García et al. 2017).

**Fig. 1:**
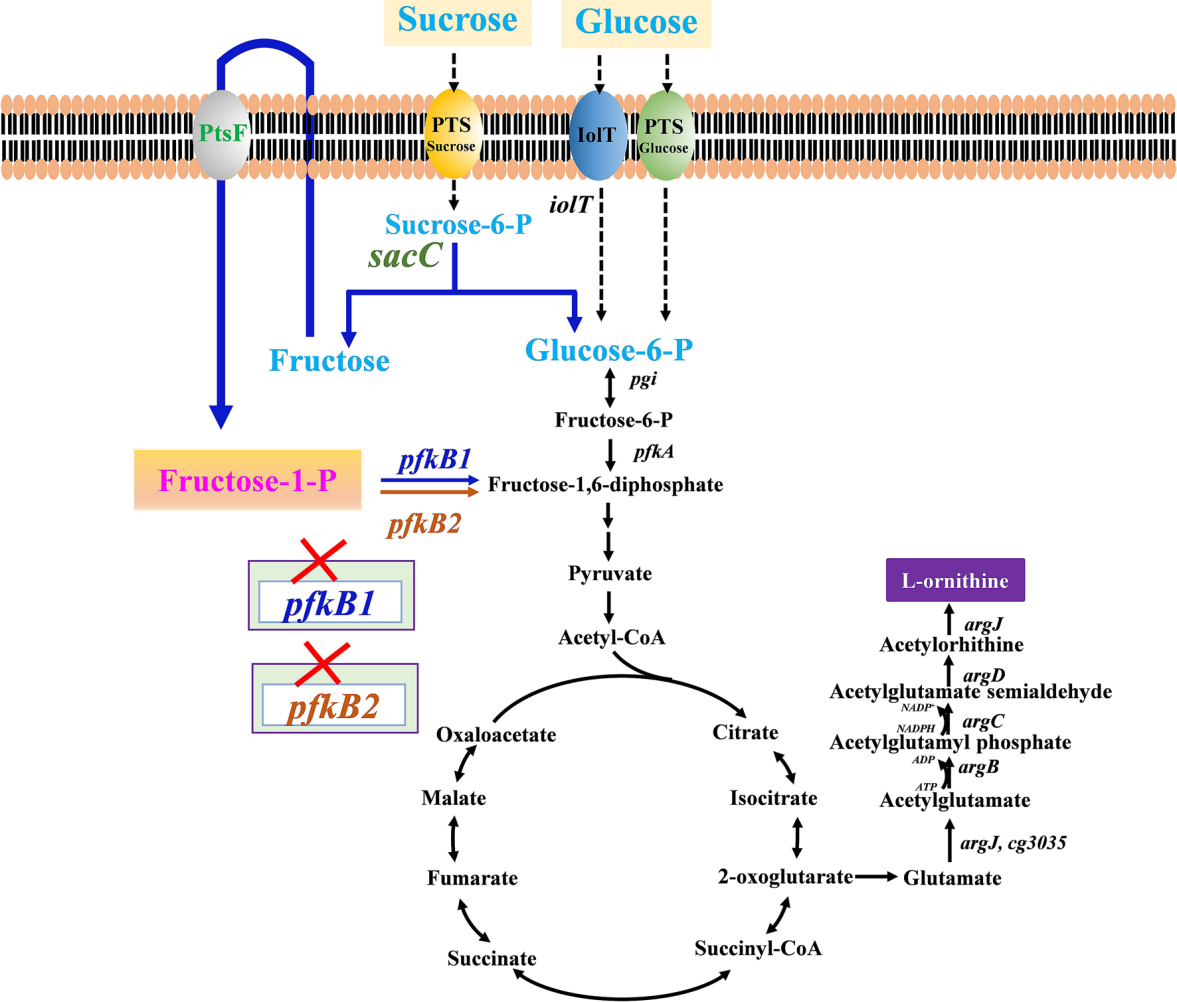
The metabolic pathways and schematic diagram of genetic engineering modulations in *C. glutamicum* for ʟ-ornithine production from glucose and sucrose. The red × indicates those genes were inactivated. *pfkB1* encodes fructose-1-phosphate kinase-1; *pfkB2* encodes fructose-1-phosphate kinase-2; *ptsF* encodes fructose transporter; *sacC* encodes sucrose-6-phosphate hydrolase

The biosynthesis of l-ornithine, an intermediate metabolite in the metabolic pathway of l-arginine, requires not only abundant intracellular NADPH to provide a reducing force, but also an unobstructed central metabolic pathway to provide sufficient precursors. Theoretically, its biosynthesis in *C. glutamicum* can be promoted using carbon sources mixed with glucose and sucrose. In previous studies, systematic metabolic engineering of *C. glutamicum* S9114, including the manipulation of the main l-ornithine metabolic pathway, reinforcement of metabolic pathways supplying the necessary precursors, improvement of the cofactor supplement of NADPH and acetyl-CoA, strengthening of the central metabolic pathway, modulation of the glucose uptake system, and exploration of the fluent transport routes, was performed to generate the engineered strain SO26, which produced a final l-ornithine titer of 43.6 g/L (Jiang et al. 2020; Zhang et al. 2017, 2018a, 2018b, 2019). Here, we improved the l-ornithine production titer by co-utilizing glucose and sucrose. Elucidation of the underlying molecular mechanisms revealed that deleting *pfkB1* remarkably promoted l-ornithine production (Fig. 1).

## Materials and methods

### Experimental material

*Corynebacterium glutamicum* SO26, a highly reconstituted strain originating from *C. glutamicum* S9114 (an l-glutamate-producing strain stored at the China Center of Industrial Culture Collection [CICC] and is denoted as CICC 20935), was adopted as the parent strain (Zhang et al. 2019). *Escherichia coli* DH5α is a popular cloning host for the construction of recombinant plasmids. Table 1 lists the engineered strains and plasmids used herein. Chemical standards were purchased from Shanghai Macklin Biochemical Technology Co., Ltd. (Shanghai, China).

**Table 1:** Strains, plasmids, and primers used in this study

Strain/plasmid/primers	Characteristic/sequence (5'–3')	Source
Strain		
* E. coli* DH5ɑ	Clone host strain	Transgen
* C. glutamicum* S9114	Industrial strain for glutamate production	
SO26	l-Ornithine producing strain derived from *C. glutamicum* S9114	(Zhang et al. 2019)
SO30	SO26 with *pfkB1* deletion	This study
SO31	SO26 with *pfkB2* deletion	This study
SO32	SO26 with *pfkB1* deletion and *pfkB2* deletion	This study
Plasmid		
pK18*mobsacB*	Mobilizable vector, allows for selection of double-crossover in *C. glutamicum*, Km^R^, *sacB*	Lab stock
pK18−△*pfkB1*	A derivative of pK18*mobsacB*, harboring △*pfkB1* fragment	This study
pK18−△*pfkB2*	A derivative of pK18*mobsacB*, harboring △*pfkB2* fragment	This study
Primers		
* pfkB1*-up-F	aacgacggccagtgccaagcttTCAGCGAGGTTAAGCATGGTC	This study
* pfkB1*-up-R	GCGTGGAATCAATGTGATGATCATGGGGTTACC	This study
* pfkB1*-down-F	CATGATCATCACATTGATTCCACGCTGTCGCTCG	This study
* pfkB1-*down-R	cggtacccggggatcctctagaCAGCTCCAACGGTGGATACAAC	This study
* pfkB1-*check-F	ACATTCACCCCAAACCCGAGT	This study
* pfkB2*-up-F	aacgacggccagtgccaagcttGATTGCCTCATTAACGGCAGT	This study
* pfkB2*-up-R	CTGCCGGATTTTGTTCCGTCAAGCTCATTGGTGCTC	This study
* pfkB2*-down-F	ATGAGCTTGACGGAACAAAATCCGGCAGGTTTCC	This study
* pfkB2-*down-R	cggtacccggggatcctctagaAATTCACGCCCTGCTTACCAG	This study
* pfkB2-*check-F	GCACCATCGAAATTGGCGAAG	This study

Superscript ‘‘R’’ indicates resistance to the following antibiotics: Km kanamycin

### Molecular cloning and strain breeding

Homologous recombination mediated by the suicide plasmid pK18*mobsacB* has been used to delete genes in *C. glutamicum* (Schäfer et al. 1994). We deleted phosphofructokinase B (*pfkB1)* by amplifying and splicing the upstream and downstream arms to generate a homologous arm that was inserted into the suicide plasmid pK18*mobsacB*. The recombinant vector pK18-∆*pfkB1* generated using a one-step cloning strategy served as a gene-targeting plasmid. We transformed pK18-∆*pfkB1* into the original *C. glutamicum* SO26 strain by electroporation, then identified single crossover strains that thrived on media containing kanamycin by polymerase chain reaction (PCR) amplification using specific primers (Table 1).

Positive single crossover strains were cultivated in antibiotic-free nutrient medium and then coated onto plates containing 10% sucrose to breed double-crossover strains (Zhang et al. 2017). Positive double-crossover strains were identified by PCR amplification using specific primers. We deleted *pfkB2* using the same strategy. All primers used for the construction of the *pfkB1* or *pfkB2* deletion strain were synthesized at Kingsley Biological Technology Co., Ltd. (Nanjing, China). If necessary, 50 and 12.5 mg/L of kanamycin were applied to propagate *E. coli* and *C. glutamicum*, respectively.

### Fermentation conditions and operation

We investigated the performance of the strains using batch cultivation in shake flasks and fed-batch cultivation in a 5-L fermenter as described with slight modifications (Zhang et al. 2019). After transfer culture twice in plates, two rings of strain sludge were inoculated into 10 mL of Luria–Bertani (LB) liquid culture medium containing 2% glucose (LBG) and shaken at 250 rpm for 12 h at 32 °C. Thereafter, 2 mL of culture was inoculated into 8 mL of seed medium prepared as described (Zhang et al. 2019) and cultivated for 12 h. Thereafter, 4 mL of culture was inoculated into fermentation medium prepared as described (Zhang et al. 2019) in 250-mL Erlenmeyer flasks with 20 mL of loaded liquid for batch cultivation. Fed-batch cultivation proceeded in a 5-L jar fermenter containing 2.7 L of fermentation medium (per liter: 30 g glucose, 30 g sucrose, 6 g yeast extract, 50 g (NH_4_)_2_SO_4_, 3 g MgSO_4_∙7H_2_O, 1 g KH_2_PO_4_, 0.5 g K_2_HPO_4_, 0.5 g Na_2_HPO_4_, 0.02 g MnSO_4_·H_2_O, and 0.02 g FeSO_4_·7H_2_O). The feeding solution contained (per liter) 300 g glucose, 300 g sucrose, 5 g yeast extract, 5 g (NH_4_)_2_SO_4_, and 1 g MgSO_4_·7H_2_O, and 300 mL of seed culture. The temperature, pH, and air-flow rate throughout fermentation were 32 °C, 6.8, and 2 L/min, respectively. Levels of dissolved oxygen were maintained at 30% by dynamic adjustment of the stirring speed. The pH was maintained at 6.8 by adding ammonium hydroxide.

### Analysis of samples collected during fermentation

The batch and fed-batch cultivation processes were, respectively, sampled every 12 and 4 h. The optical density at 600 nm (OD_600_) was measured using a Waters microplate reader to monitor cell growth (Waters Corp, Milford, MA, USA). The concentration of l-ornithine was measured colorimetrically as described (Jiang et al. 2013; Rosen 1957). Glucose, sucrose, and fructose were measured by high-performance liquid chromatography using a Prevail™ Carbohydrate ES column (250 × 4.6 mm, 5 μm; Alltech Associates Inc., Deerfield, IL, USA) at 35 °C and a refractive index detector at 40 °C. The sample injection volume was 10 μL, and the mobile phase was acetonitrile: water (75:25) at a flow rate of 1 mL/min.

## Results and discussion

### Glucose and sucrose co-utilization for l-ornithine production

**Fig. 2:**
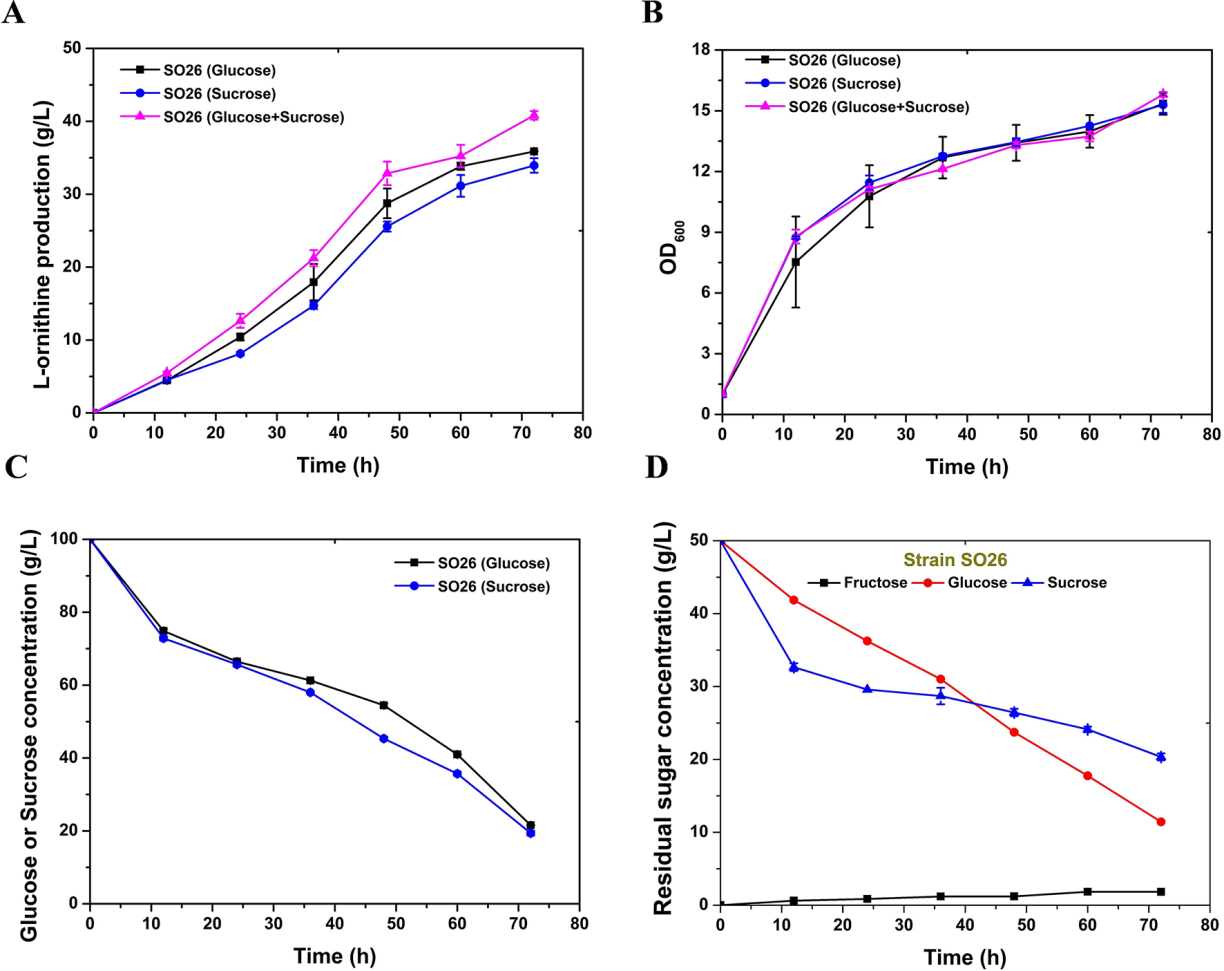
Shake-flask fermentation evaluation of *C. glutamicum* SO26 on different carbon sources. **A** l-Ornithine concentration in the fermentation liquids of *C. glutamicum* SO26 cultivated on glucose (solid black square), sucrose (solid blue cycle), and glucose plus sucrose (solid pink triangle). **B** Cell growth of *C. glutamicum* SO26 cultivated on glucose (solid black square), sucrose (solid blue cycle), and glucose plus sucrose (solid pink triangle). **C** Sugar consumption of *C. glutamicum* SO26 cultivated on glucose (solid black square), sucrose (solid blue cycle). **D** Sugar consumption of *C. glutamicum* SO26 cultivated on glucose plus sucrose. Black square represents fructose, red cycle represents glucose, and blue triangle represents sucrose. Data are averages and standard deviations from three independent experiments

l-Ornithine is an intermediate metabolite in the l-arginine biosynthesis metabolic pathway. In a previous study, the mixed utilization of glucose and sucrose resulted in a marked improvement in l-arginine production in *C. glutamicum* (Park et al. 2014). In theory, the utilization of a mixture of glucose and sucrose is expected to increase l-ornithine production as improving l-arginine accumulation in *C. glutamicum*. To test this hypothesis, we conducted fermentation assays of the engineered strain *C. glutamicum* SO26, which showed the best l-ornithine production performance in our laboratory, using different carbon sources. During 72 h of batch cultivation, 40.82 g/L of l-ornithine was produced using isometric glucose and sucrose (1:1 weight ratio), which represents a 13.8% increase in the production titer compared to using glucose as the sole carbon source (Fig. 2A, Table 2). These findings confirmed that glucose and sucrose co-utilization significantly promotes l-ornithine accumulation which further indicates that the production of l-glutamate family chemicals could be improved by using glucose and sucrose as carbon sources. However, the yield of l-ornithine obtained using sucrose as the sole carbon source was only 33.96 g/L, which was lower than that produced by glucose alone or in combination with sucrose (1:1 w/w; Fig. 2A, Table 2). This result was consistent with that of a previous study in which the l-arginine production titer declined when sucrose was the sole carbon source (Park et al. 2014).

**Table 2:** Engineering *C. glutamicum* for biobased l-ornithine production driven by glucose and sucrose

Strains (*C. glutamicum*)	Carbon sources	l-Ornithine accumulation (g/L)	Cell biomass (OD_600_)	l-Ornithine/OD600	Glucose concentration (g/L)	Sucrose concentration (g/L)	Fructose concentration (g/L)
SO26	Glucose	35.88 ± 0.04	11.68 ± 0.43	3.07	21.54 ± 0.09	–	–
	Sucrose	33.96 ± 1.00	12.90 ± 0.22	2.63	2.54 ± 0.01	19.37 ± 0.60	1.62 ± 0.03
	Glucose + sucrose	40.82 ± 0.58	12.91 ± 0.20	3.16	11.43 ± 0.29	20.33 ± 0.47	1.82 ± 0.01
SO30	Glucose	39.08 ± 1.22	14.31 ± 0.33	2.73	–	–	–
	Glucose + sucrose	47.64 ± 0.18	12.77 ± 0.10	3.73	3.59 ± 0.23	21.60 ± 1.17	2.87 ± 0.01
SO31	Glucose	38.85 ± 1.56	13.24 ± 0.46	2.93	–	–	–
	Glucose + sucrose	41.91 ± 0.90	12.69 ± 0.03	3.30	14.09 ± 0.35	19.74 ± 0.46	1.68 ± 0.03
SO32	Glucose + sucrose	32.74 ± 0.21	14.17 ± 0.21	2.31	2.02 ± 0.25	21.05 ± 0.02	11.18 ± 0.27
SO30 (Fed-batch)	Glucose + sucrose	78.0	14.53	5.37	0	13.39	34.23

Fermentations were performed at 250 rpm for 72 h, and the initial sugar concentration was 100 g/L. Results except fed-batch cultivation are the means ± standard deviations in three individual experiments

The growth of *C. glutamicum* SO26 cultivated on glucose and sucrose (1:1 w/w) and on sucrose was slightly higher than that obtained with glucose as the sole carbon source (Fig. 2B). The improved cell growth suggests that *C. glutamicum* SO26 prefers sucrose as a carbon source (Fig. 2C), which was contrary to the previous findings of a decline in cell growth (Park et al. 2014). This inconsistency in the cell growth response to sucrose might have been due to variations among strains with different genetic traits. *C. glutamicum* SO26 is more sensitive to sucrose and consumes it faster when it is the sole carbon source (Fig. 2D). Compared with other *C. glutamicum* strains, SO26 showed less escape during sucrose reverse screening of positive double-crossover recombinant strains. However, accelerated cell growth was not accompanied by a better yield of l-ornithine. We considered that because glucose was the carbon source for all previous genetic manipulations of *C. glutamicum* SO26, less optimization of the sucrose and fructose metabolic pathways resulted in lower l-ornithine production from sucrose than from glucose as the carbon source.

### Deleting *pfkB1* improved l-ornithine production in *C. glutamicum*

**Fig. 3:**
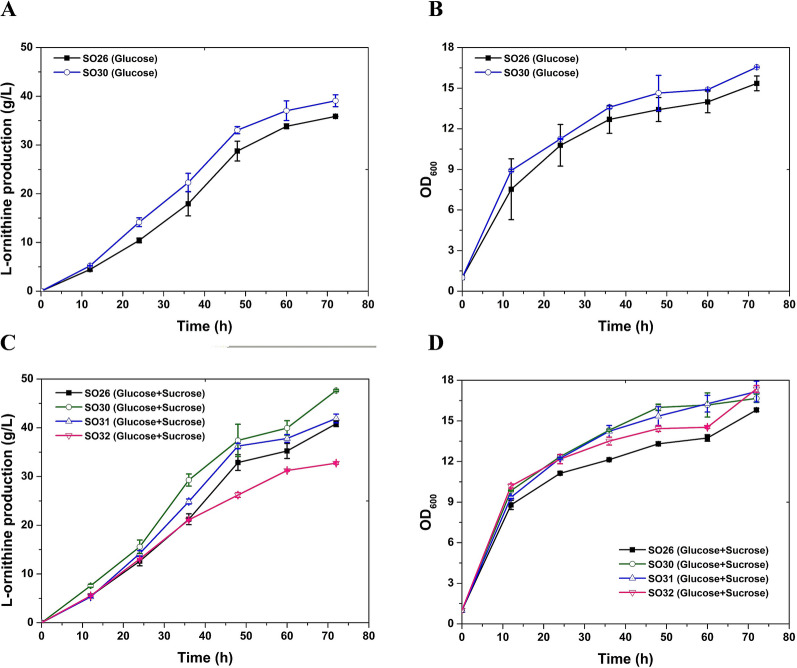
Manipulation of fructose metabolic pathway promotes l-ornithine accumulation in *C. glutamicum* SO26. **A** l-Ornithine production curves of strain *C. glutamicum* SO26 (solid black square) and SO30 (with *pfkB1* deletion, hollow blue cycle) cultivated on glucose. **B** Cell growth curves of strain *C. glutamicum* SO26 (solid black square) and SO30 (hollow blue cycle) cultivated on glucose. **C** l-Ornithine concentration in the fermentation liquids of strain SO26 (solid black square), SO30 (hollow green cycle), SO31 (hollow blue upper triangle), and SO32 (hollow red lower triangle) cultivated on glucose plus sucrose. **D** Cell growth of strain SO26 (solid black square), SO30 (hollow green cycle), SO31 (hollow blue upper triangle), and SO32 (hollow red lower triangle) cultivated on glucose plus sucrose. Data are averages and standard deviations from three independent experiments

**Fig. 4:**
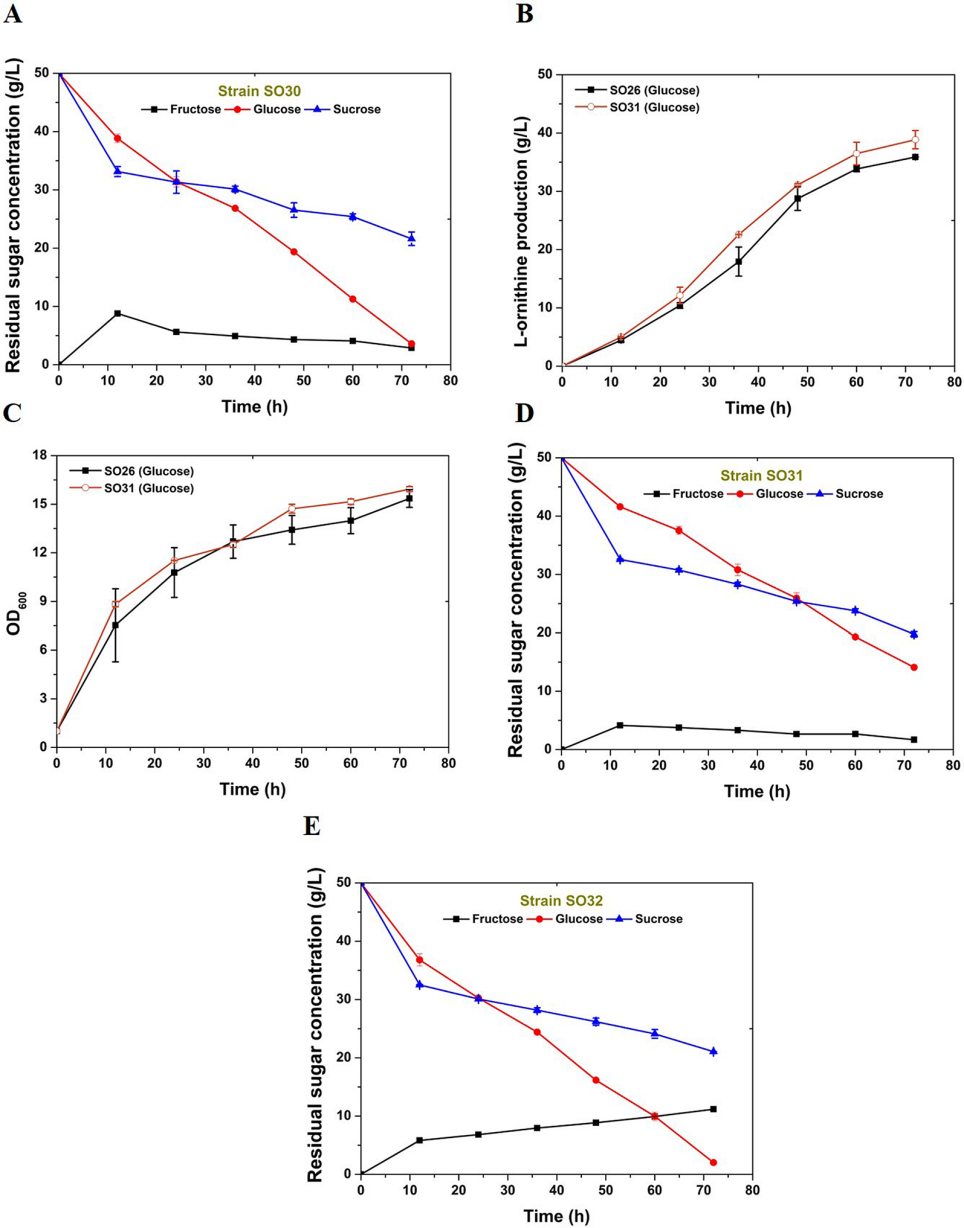
Deletion of *pfkB2* promotes l-ornithine accumulation in *C. glutamicum* SO26. **A** Sugar consumption of *C. glutamicum* SO30 cultivated on glucose plus sucrose. Black square represents fructose, red cycle represents glucose, and blue triangle represents sucrose. **B** l-Ornithine production curves of strain *C. glutamicum* SO26 (solid black square) and SO31 (with *pfkB2* deletion, hollow red cycle) cultivated on glucose. **C** Cell growth curves of strain *C. glutamicum* SO26 (solid black square) and SO31 (hollow red cycle) cultivated on glucose. **D** Sugar consumption of *C. glutamicum* SO31 cultivated on glucose plus sucrose. Black square represents fructose, red cycle represents glucose, and blue triangle represents sucrose. **E** Sugar consumption of *C. glutamicum* SO32 cultivated on glucose plus sucrose. Black square represents fructose, red cycle represents glucose, and blue triangle represents sucrose. Data are averages and standard deviations from three independent experiments

As discussed above, mixed utilization of glucose and sucrose to cultivate C. *glutamicum* SO26 exerts a positive effect on the biosynthesis of l-ornithine. In addition, as claimed in previous studies, introducing sucrose into the fermentation medium of C. *glutamicum* increases the intracellular abundance of NADPH, glucose phosphate, and fructose phosphate, which subsequently promotes the biosynthesis of various important metabolites (Wang et al. 2016). In C. *glutamicum* SO26, a multitude of gene operations, including attenuation of glucose-6-phosphate isomerase expression in the glycolytic pathway and the overexpression of enzymes in the pentose phosphate or tricarboxylic acid cycle pathways provide sufficient cofactors for l-ornithine biosynthesis. Therefore, we speculated that the accumulation of intracellular fructose-1-phosphate, which stimulates upregulation of the corresponding genes in glycolysis that accelerate glucose consumption (Hasegawa et al. 2017), is the primary reason why the l-ornithine production titer was improved when glucose and sucrose comprised the carbon source. To test this hypothesis, we disrupted the *pfkB1* gene encoding fructose-1-phosphate kinase to prevent fructose-1-phosphate catabolism and generated *C. glutamicum* SO30. Subsequently, we evaluated the performance of this engineered strain using shake-flask fermentation with glucose alone or mixed with sucrose (1:1 w/w) as the carbon source. Figure 3A and Table 2 show that *C. glutamicum* SO30 produced 39.08 g/L of l-ornithine from glucose at 72 h, which was 8.9% more than that produced by the control strain *C. glutamicum* SO26. The cell growth of strain *C. glutamicum* SO30 also improved slightly compared with that of the control strain *C. glutamicum* SO26 (Fig. 3B, Table 2). Deleting *pfkB1* increased the l-ornithine production titer by~ 16.7%, from 40.82 to 47.64 g/L, when cultivated with glucose and sucrose (1:1 w/w; Fig. 3C, Table 2). Homoplastically, the cell growth of *C. glutamicum* SO30 was remarkably improved throughout fermentation compared with that of the control strain *C. glutamicum* SO26 (Fig. 3D). Glucose utilization was accelerated and fructose accumulation was not excessive in SO30 with the *pfkB* deletion compared with that in SO26 (Fig. 4A). The remarkable improvement in l-ornithine yield realized by deleting *pfkB1* suggested that fructose-1-phosphate accumulation benefits l-ornithine biosynthesis. When cultivated on glucose and sucrose, significantly increased l-ornithine production titer indicating that l-ornithine accumulation correlates positively with accelerated glucose utilization.

### Deleting *pfkB2* improved l-ornithine production in *C. glutamicum*

Fructose accumulation was not excessive in the fermentation supernatant of *C. glutamicum* SO30, indicating that deleting *pfkB1* did not block the fructose utilization pathway. Analysis of the genome of *C. glutamicum* S9114, the parent strain of *C. glutamicum* SO26, revealed another fructose phosphokinase (encoded by *pfkB2*) that catalyzed the conversion of fructose-1-phosphate to fructose-1,6-diphosphate. These two enzymes are believed to catalyze the fructose utilization pathway in *C. glutamicum* SO26, and the deletion of *pfkB2* is presumed to promote l-ornithine accumulation. To test this hypothesis, we engineered *C. glutamicum* SO31 with inactivated *pfkB2* and evaluated its performance in shake-flask fermentation. The results were similar to those of *C. glutamicum* SO30. When cultivated on glucose, *C. glutamicum* SO31 produced 38.85 g/L of l-ornithine, which was an 8.3% increase compared with that produced by the parent strain *C. glutamicum* SO26. The growth of *C. glutamicum* SO31 was also slightly improved compared with that of *C. glutamicum* SO26 (Fig. 4B and C; Table 2). When cultivated on glucose and sucrose (1:1 w/w), *C. glutamicum* SO31 produced 41.91 g/L of l-ornithine at 72 h, which was comparable to that produced by *C. glutamicum* SO26. However, the l-ornithine yield significantly increased during the early phase of fermentation, indicating that the *pfkB2* deletion also promoted the biosynthesis of l-ornithine in *C. glutamicum* SO26 (Fig. 3C). Compared with strain SO31, *pfkB1* inactivation generated a relatively higher l-ornithine production titer in *C. glutamicum* SO30, which might have been due to its major role in the conversion of fructose-1-phosphate to fructose-1,6-diphosphate. The growth of *C. glutamicum* SO31 was slightly better than that of its parent strain, which was consistent with the results obtained when *pfkB1* was deleted (Fig. 4C). Strain SO31 consumed similar amounts of glucose and sucrose and increased fructose accumulation as compared with the original strain SO26 (Fig. 4D). However, less fructose accumulated in SO31 than in SO30 (Fig. 4A), indicating the predominant function of *pfkB1* in the catalyzation from fructose-1-phosphate to fructose-1,6-diphosphate. In summary, these results further confirmed our hypothesis that deleting fructose phosphokinase causes the accumulation of intracellular fructose-1-phosphate, which favors l-ornithine biosynthesis in *C. glutamicum*.

### Effects of double deleting *pfkB1* and *pfkB2* on l-ornithine production

The present findings suggested that deleting either *pfkB*1 or *pfkB2* promotes l-ornithine accumulation. However, whether these two targets have synergistic effects and whether there is a third pathway for the conversion of fructose-1-phosphate to fructose-1,6-diphosphate have remained controversial. To cope with these issues, we therefore deleted *pfkB*1 and *pfkB2* in *C. glutamicum* SO26 to generate *C. glutamicum* SO32 and then evaluated its performance in shake-flask fermentation with glucose and sucrose as carbon sources. Batch cultivation for 72 h produced 32.74 g/L of l-ornithine, which was 19.8%, 31.3%, and 21.9% lower than the production titer of SO26, SO30, and SO31, respectively (Fig. 3C, Table 2). The growth of *C. glutamicum* SO32 also slightly increased compared with that of the control SO26 strain (Fig. 3D). Moreover, the engineered *C. glutamicum* SO32 accumulated 11.18 g/L of fructose in the fermentation supernatant (Fig. 4E, Table 2). We inferred from these results that deleting *pfkB*1 and *pfkB2* reduced l-ornithine production by almost completely blocking the fructose utilization pathway. In practice, the co-utilization of fructose contributes to the biosynthesis of l-ornithine by *C. glutamicum* cultivated with glucose and sucrose (1:1 w/w) as carbon sources. Compared with the deletion of *pfkB1* and *pfkB2*, deleting only *pfkB1* in *C. glutamicum* not only enabled the accumulation of intracellular fructose-1-phosphate and accelerated the glucose utilization pathway, but also drove the conversion of fructose to l-ornithine. These results indicated that this is an ideal strategy for engineering strains to produce l-ornithine.

### Fed-batch fermentation of engineered *C. glutamicum* SO30

**Fig. 5:**
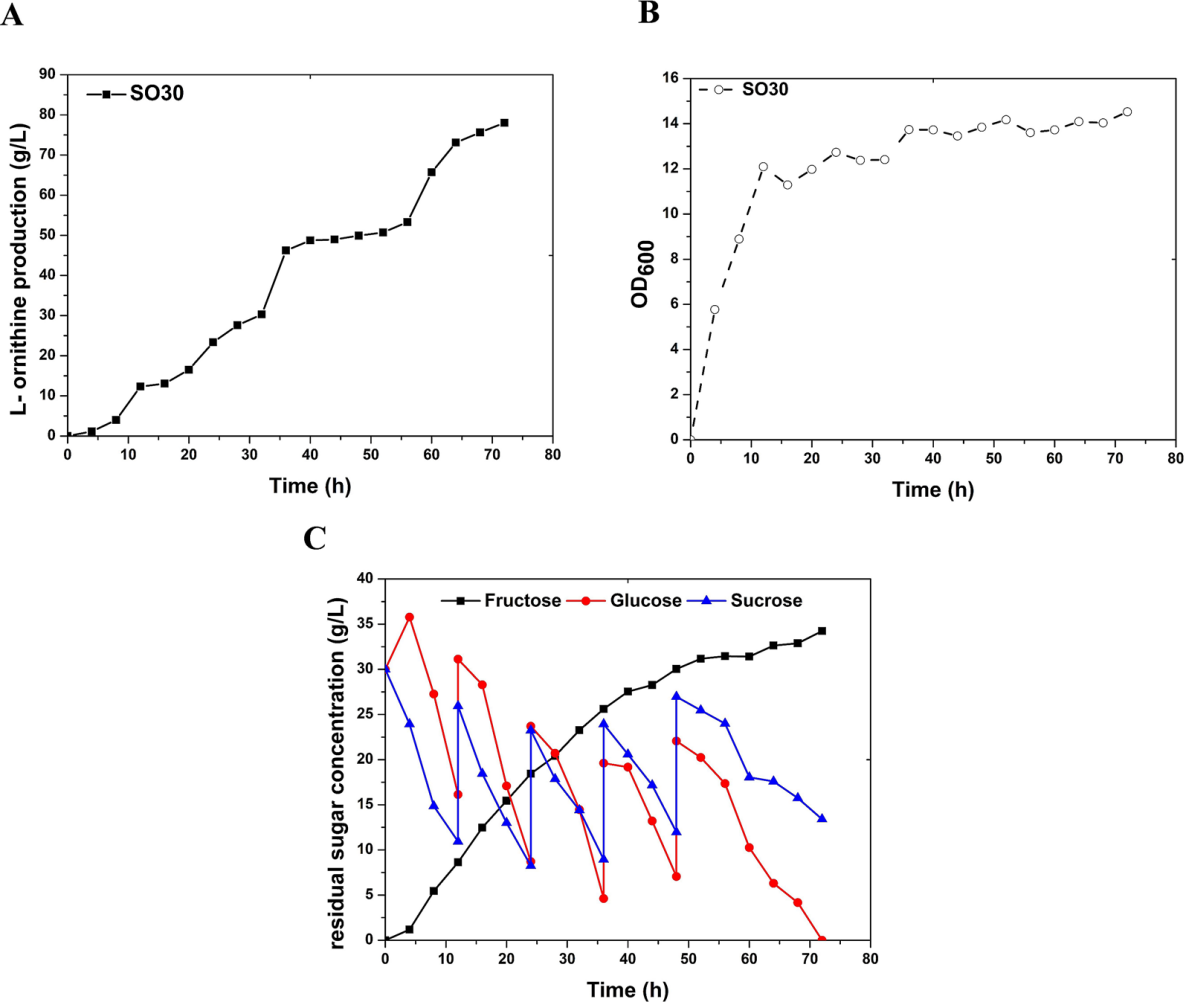
Fed-batch fermentation evaluation of *C. glutamicum* SO30 in a 5-L fermenter. **A** l-Ornithine production curves of strain SO30 in fed-batch cultivation. **B** Cell growth curves of strain SO30 in fed-batch cultivation. **C** Glucose (solid red cycle), sucrose (solid blue triangle), and fructose (solid black square) concentrations in the fermentation liquids

To investigate the possibility of further enhancing the l-ornithine yield, we performed using fed-batch fermentation of the engineered strain *C. glutamicum* SO30 in a 5-L fermenter for 72 h using a mixture of glucose and sucrose (1:1 w/w) as carbon sources. Fermentation results showed that *C. glutamicum* SO30 produced 78.0 g/L of l-ornithine with a total glucose and sucrose yield of 0.52 g/g (Fig. 5A, B and C; Table 2). Compared with our previous results (43.6 g/L) (Zhang et al. 2019), *C. glutamicum* SO30 produced 78.9% more l-ornithine from a mixture glucose and sucrose as carbon sources. The deletion of *pfkB1* remarkably promoted l-ornithine biosynthesis, rendering this strategy applicable to the production of other valuable metabolites, such as l-citrulline, l-lysine, l-proline, and l-arginine.

## Conclusion

This study is the first to demonstrate the positive effects of glucose and sucrose as the carbon sources on l-ornithine production. *Corynebacterium glutamicum* SO30 generated a final l-ornithine titer of 78 g/L, which represents a significant improvement over that generated by *C. glutamicum* SO26. The metabolic engineering strategy of disrupting *pfkB1* to accelerate glucose utilization pathways provides a powerful guide for the development of engineered *C. glutamicum* to produce valuable chemicals.

## Data Availability

Data sharing is not applicable to this article as no datasets were generated or analyzed during the current study.

## Abbreviations

LB: Luria–Bertani
NADPH: Nicotinamide adenine dinucleotide phosphate
